# A rare complication of brucellosis: Superinfection of a mature ovarian cystic teratoma

**DOI:** 10.1590/0037-8682-0521-2023

**Published:** 2024-02-05

**Authors:** Ömer Karaşahin, Orkun Ilgen, Emine Füsun Karaşahin

**Affiliations:** 1 Erzurum City Hospital, Department of Infectious Diseases and Clinical Microbiology, Erzurum, Turkey.; 2 Erzurum City Hospital, Department of Gynecologic Oncology, Erzurum, Turkey.; 3 Erzurum Provincial Health Directorate, Department of Public Health, Erzurum, Turkey.

**FIGURE 1:**
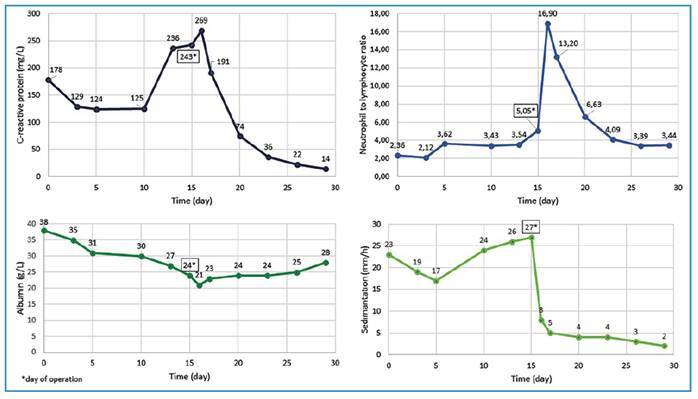
Changes in selected inflammatory markers during the course of treatment.

**FIGURE 2:**
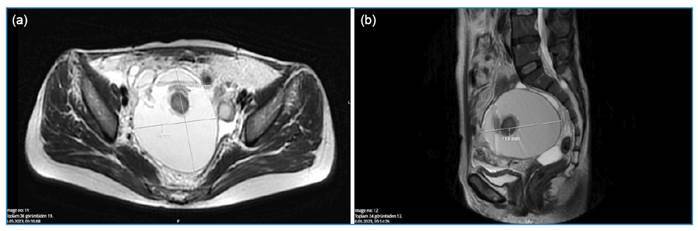
Mature ovarian cystic teratoma on T2-weighted axial **(a)** and sagittal **(b)** magnetic resonance imaging.

**FIGURE 3:**
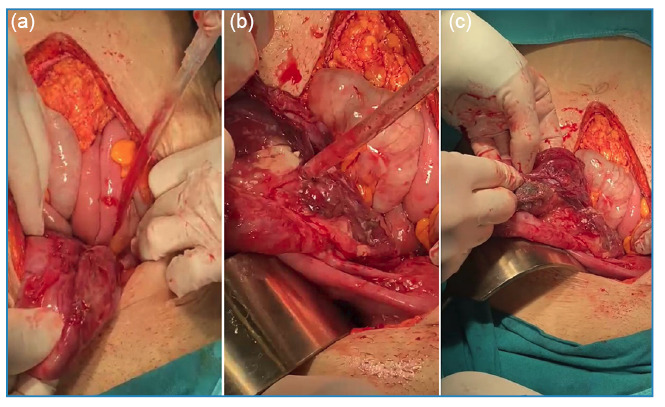
Intraoperative images of the ovarian mature cystic teratoma **(a)**, showing the presence of adipose tissue **(b)**, and hair **(c)** in the cyst.

A 43-year-old woman rural dweller working in animal husbandry presented with high fever, chills, nausea, and vomiting. She was diagnosed with brucellosis based on the *Brucella* standard tube agglutination test and blood culture results. Due to persistent vomiting, she was treated with intravenous tigecycline and oral gentamicin and rifampicin therapy. However, despite appropriate antimicrobial treatment, a clinical and laboratory response was not achieved. The course of inflammation indicators during treatment is shown in Figure 1. Right lower quadrant tenderness was noted on abdominal examination. Magnetic resonance imaging revealed a 12-cm cyst in the region of the right ovary, which was suspected to be a mature cystic teratoma (Figure 2). We performed right ovariectomy and cystectomy (Figure 3). The patient’s fever, nausea, and vomiting resolved from postoperative day 2. İntraoperative cultures of samples of the teratoma yielded *Brucella* spp. The patient's treatment was switched to rifampicin and doxycycline 2 weeks postoperatively and was discontinued after 8 weeks. Treatment failure in brucellosis is defined as the persistence of disease signs and/or symptoms after the initiation of appropriate antimicrobial therapy^1^. The most common complications of brucellosis are osteoarticular and genitourinary involvement^2^. Considering the potential of *Brucella* to invade several tissues, this case illustrates the importance of genitourinary system evaluation in the event of treatment failure^3^. Furthermore, tigecycline may be an option for combination therapy in patients with severe brucellosis^4^.
